# Supplementary material for: Vascular Remodeling Is a Crucial Event in the Early Phase of Hepatocarcinogenesis in Rodent Models for Liver Tumorigenesis
Source: Cells. 2022 Jul 6;11(14):2129. doi: 10.3390/cells11142129 (PMC9320355; doi:10.3390/cells11142129)
Supplement: Supplementary file 1 [file cells-11-02129-s001.zip › cells-1767626-supplementary.pdf]

cells MDPI

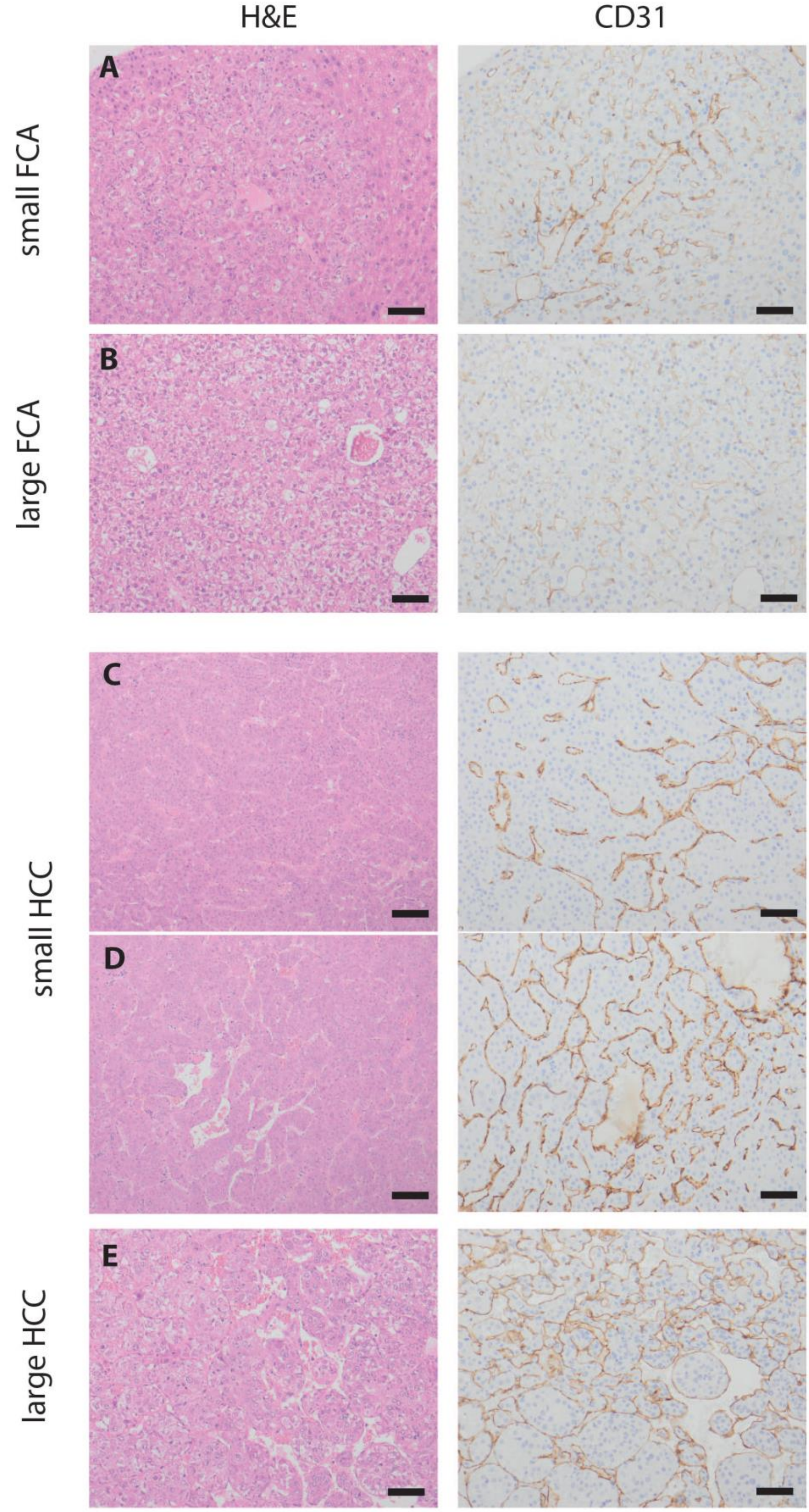

Figure S1. Representative images of the H&E based vascular morphology in small and large Foci of cellular alteration (FCA). While FCA (**A**,**B**) present with a weak staining in CD31 showing narrow vessels with slight branching, HCC vasculature presented with stronger staining in CD31 highlighting different vascular pattern: elongated vessels with branching surrounding larger tumor clusters (**C**), with focal dilatation (**D**) or large dilated vessels in macrotrabecular HCC areas (**E**). Scale bar: 50 μm (magnification 20×).

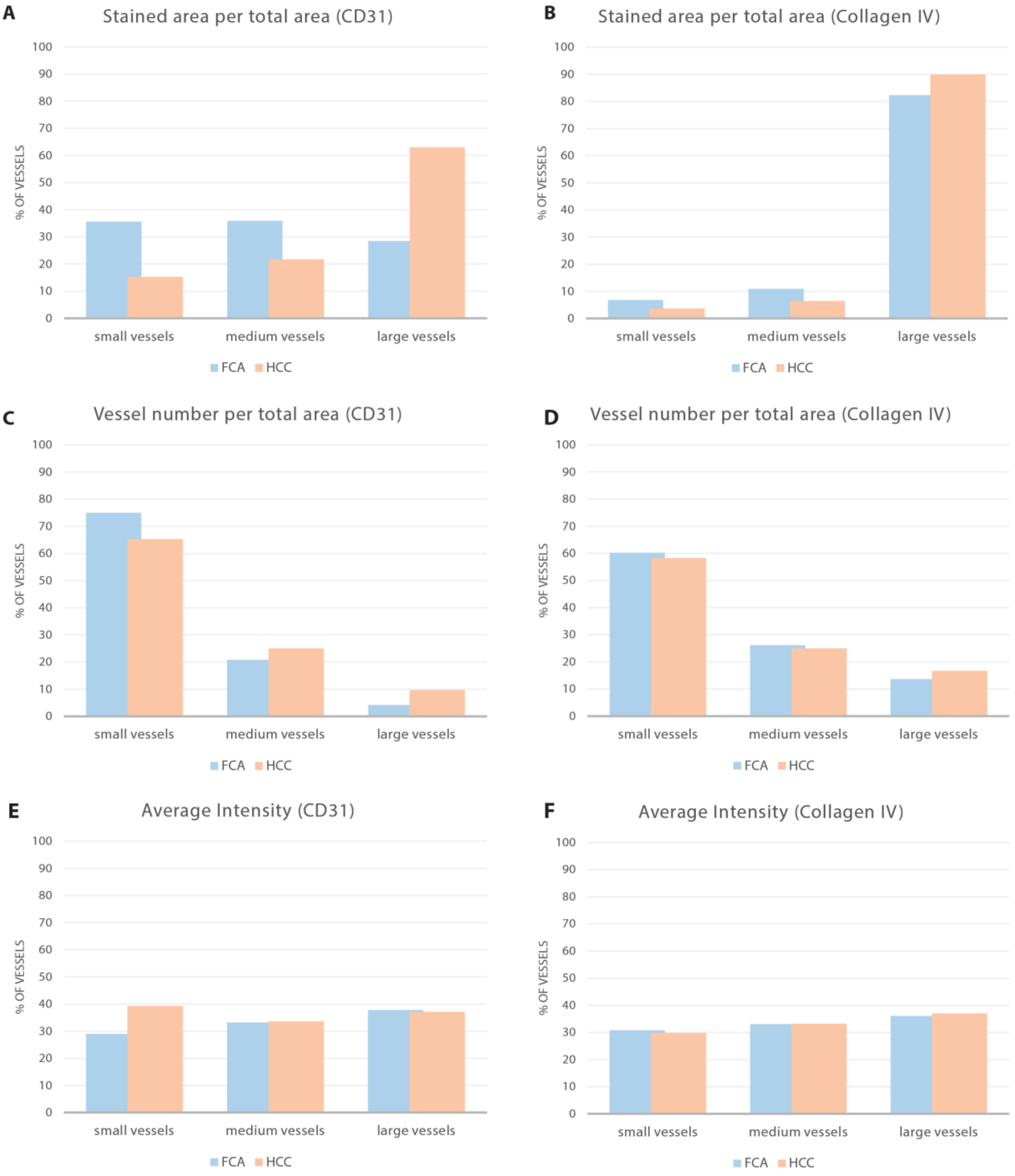

Figure S2. The distribution of vessels according to size subgroups and investigated parameter: (**A**,**B**): stained area per total area (left panel: CD31, right panel Collagen IV). (**C**,**D**): vessel number per total area (left panel: CD31, right panel Collagen IV). (**E**,**F**): average staining intensity (left panel: CD31, right panel Collagen IV). (**A**,**C**,**E**) CD31; (**B**,**D**,**F**) Collagen IV.

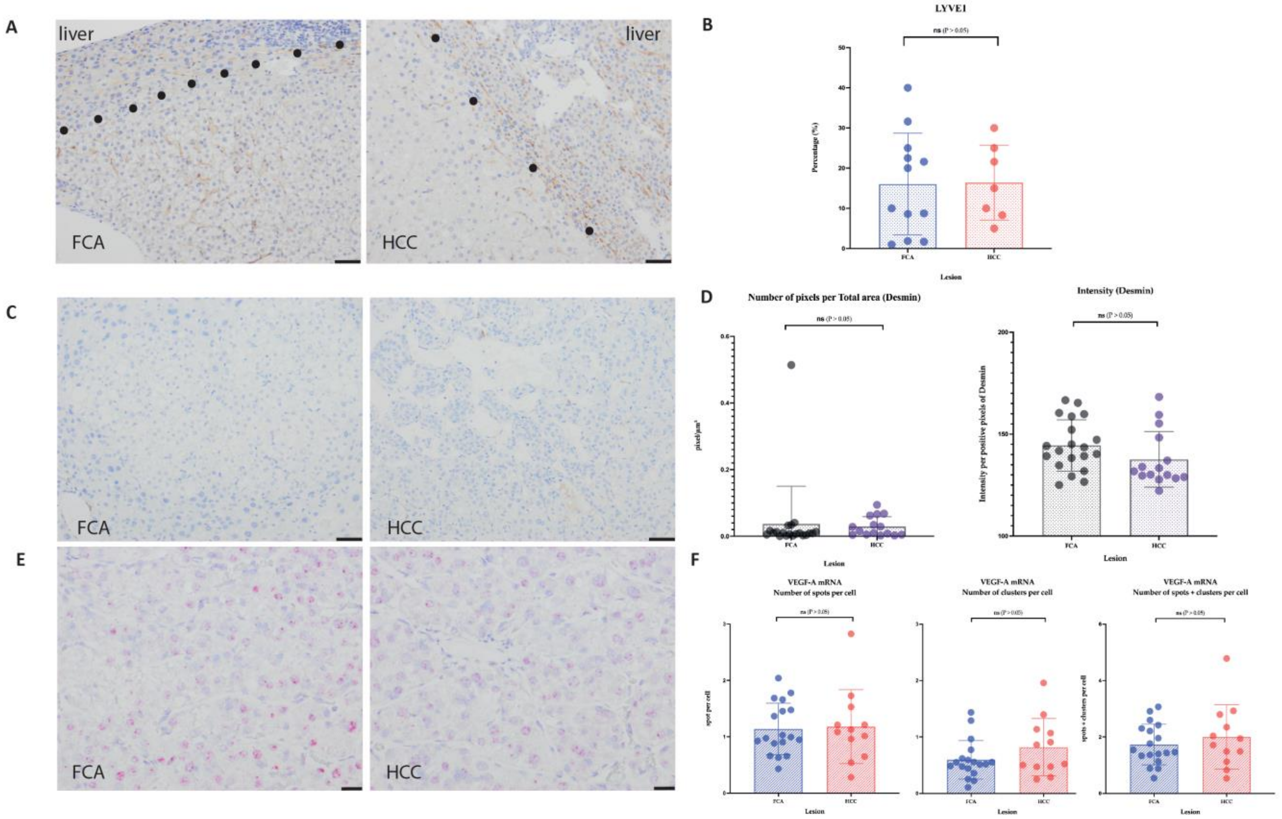

Figure S3. Analysis of vessels per lesion for expression of LYVE, Desmin and VEGF mRNA. (A) Immunostainings of LYVE in FCA and HCC (**A**) showing a weak staining equal or less compared to sinusoidal expression of surrounding liver. (**B**) Semi-quantative analysis of percentage of LYVE positive vessels in FCA and HCC. (**C**) Immunostainings of Desmin in FCA and HCC, (**D**) number of Desmin positive pixel/total area (left panel) and intensity of Desmin/positive pixel (right panel). (**E**) mRNA in situ hybridization of VEGF-A in FCA and HCC, (**F**) Number of VEGF positive spots and clusters per cell (left panel), Number of VEGF positive spots per cell (center), (**J**) Number of VEGF positive clusters per cell (right panel). Error bars indicate mean and standard deviation for each lesion. *p*-values: Not statistically significant (ns) p value > 0.05, for statistical significance accepted p value ≤ 0.05. *p value ≤ 0.05, **p value ≤ 0.01, ***p value ≤ 0.001, ****p value ≤ 0.0001. Scale bars: (**A**,**C**): 50µm (20× magnification), (**E**): 20µm (40× magnification).

A

General observations						
	Total number of vessels		Average staining intensity of vessels		Total stained vessel area	
	FCA	HCC	FCA	HCC	FCA	HCC
**CD31**	+	-	-	+	x	x
**Collagen IV**	+	-	x	x	x	x

Vessel area						
	FCA			HCC		
	Small	Medium	Large	Small	Medium	Large
**CD31**	+	+	-	-	-	+
**Collagen IV**	+	+	x	-	-	x

Vessel number						
	FCA			HCC		
	Small	Medium	Large	Small	Medium	Large
**CD31**	+	+	x	-	-	x
**Collagen IV**	+	+	+	-	-	-

Average vessel intensity						
	FCA			HCC		
	Small	Medium	Large	Small	Medium	Large
**CD31**	-	-	-	+	+	+
**Collagen IV**	x	x	x	x	x	x

B

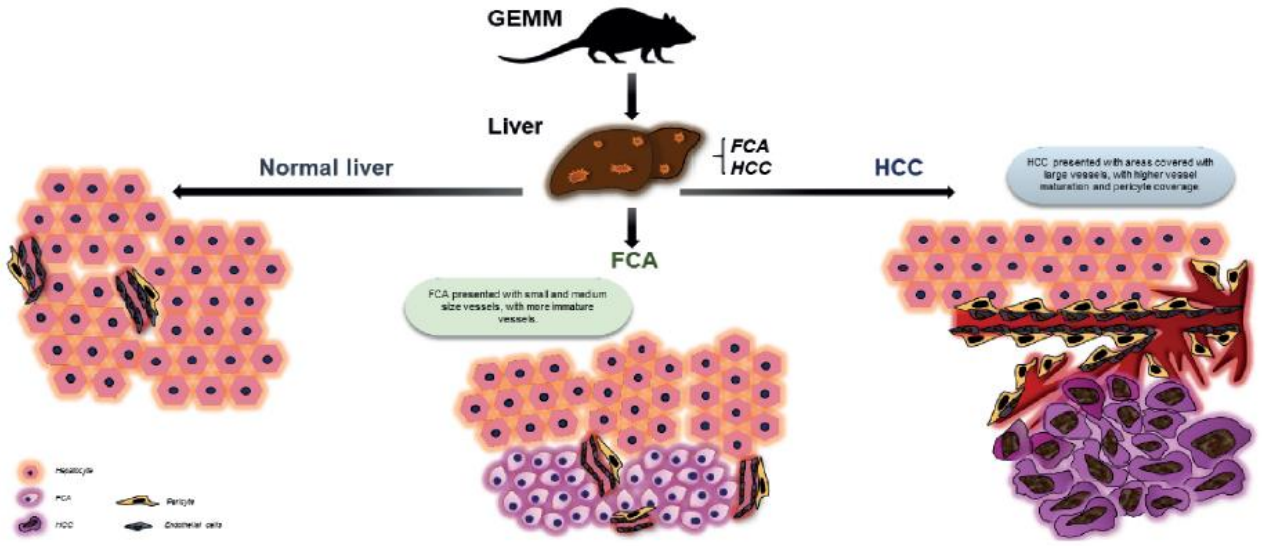

Figure S4. Simplified summary of results. (A) Simplified table with summary of all computed-based results on general observations, vessel size, number and staining intensity. X: Not statistically significant: Statistical significantly **more** was marked as "+", statistically significantly **less** was marked as "−". FCA = foci of cellular alteration, HCC = hepatocellular carcinoma. **(B)** Simplified cartoon.
